# Collision Avoidance Resource Allocation for LoRaWAN

**DOI:** 10.3390/s21041218

**Published:** 2021-02-09

**Authors:** Natalia Chinchilla-Romero, Jorge Navarro-Ortiz, Pablo Muñoz, Pablo Ameigeiras

**Affiliations:** 1 Department of Signal Theory, Telematics and Communications, University of Granada, 18071 Granada, Spain; jorgenavarro@ugr.es (J.N.-O.); pabloml@ugr.es (P.M.); pameigeiras@ugr.es (P.A.); 2 Research Center on Information and Communication Technologies, University of Granada, 18014 Granada, Spain

**Keywords:** LoRa MAC, LoRaWAN, capacity, PER, throughput, path loss

## Abstract

The number of connected IoT devices is significantly increasing and it is expected to reach more than two dozens of billions of IoT connections in the coming years. Low Power Wide Area Networks (LPWAN) have become very relevant for this new paradigm due to features such as large coverage and low power consumption. One of the most appealing technologies among these networks is LoRaWAN. Although it may be considered as one of the most mature LPWAN platforms, there are still open gaps such as its capacity limitations. For this reason, this work proposes a collision avoidance resource allocation algorithm named the Collision Avoidance Resource Allocation (CARA) algorithm with the objective of significantly increase system capacity. CARA leverages the multichannel structure and the orthogonality of spreading factors in LoRaWAN networks to avoid collisions among devices. Simulation results show that, assuming ideal radio link conditions, our proposal outperforms in 95.2% the capacity of a standard LoRaWAN network and increases the capacity by almost 40% assuming a realistic propagation model. In addition, it has been verified that CARA devices can coexist with LoRaWAN traditional devices, thus allowing the simultaneous transmissions of both types of devices. Moreover, a proof-of-concept has been implemented using commercial equipment in order to check the feasibility and the correct operation of our solution.

## 1. Introduction

According to Internet of Things (IoT) Analytics, the IoT connections are expected to grow from 7 billion in 2018 to 22 billion in 2025 [1]. In 2019, 231 million of these IoT connections came from Low Power Wide Area Networks (LPWANs) [2]. This kind of IoT wireless networks support the connection of a massive number of low-cost devices, providing long range communications that transmit small amounts of traffic but requires low energy consumption.

There are four dominant Low Power Wide Area Networks (LPWAN) technologies in the market today: LoRaWAN, Sigfox, NB-IoT and LTE-M. LoRaWAN is one of the most promising solutions which fulfills the long-range and low-power requirements, allowing bit rates between 250 bps and 11 kbps in the non-licensed ISM band. This open standard defines the medium access control (MAC) layer and the network topology. In addition, LoRaWAN has been deployed in 157 countries around the world and is backed by the LoRa Alliance, a non profit association of more than 500 members [3].

Although LoRaWAN has become one of the most popular LPWAN technologies due to its characteristics and ease of deployment, there are several weak points that the research community has identified.

One of the main limitations in LoRaWAN is the reduction of capacity due to collisions caused by the ALOHA channel access mechanism and, consequently, this aspect leads to a degradation of the overall network performance. Moreover, as depicted in [4], there is a capacity limitation due to the duty cycle regulations that limit the transmission time for each end-device in a sub-band and, also, by the number of collisions produced by the use of low data rates (DRs). So, the main challenge that we have addressed is the coordination of the resources over the ALOHA channels in order to achieve the maximum possible capacity. For this reason, this paper proposes CARA (Collision Avoidance Resource Allocation for LoRaWAN networks), a modification of the LoRaWAN MAC layer which improves network capacity by employing a smart algorithm for resource assignment.

CARA is designed to cover any kind of scenario, both rural and urban and even indoor scenarios. Specifically, as CARA exploits the LoRaWAN ALOHA channel access, it is designed for applications such as smart metering, smart agriculture, smart cities, smart homes, smart healthcare, and so forth. Based on the results obtained in the simulations, it outperforms the standard LoRaWAN channel access in urban environments of up to 8 × 8 km${}^{2}$.

The paper is structured as follows. Section 2 deals with the related work. Section 3 describes the main technical aspects about LoRa and LoRaWAN. Section 4 presents the novel LoRaWAN MAC design proposed in this paper. Section 5 details all the experimentation part and evaluates the performance of our solution by means of simulations.The coexistence in a homogeneous network with CARA and LoRaWAN traditional devices has been checked and verified in Section 6. Our solution has been implemented using commercial hardware in order to provide a proof-of-concept, which is explained in Section 7. Finally, Section 8 draws the main conclusions of this work.

## 2. Related Works

Tommaso Polonelli et al. [5] proposes an approach to increase the single channel capacity using the Slotted ALOHA protocol for the communication between end-nodes and gateways. They tested this proposal in a real environment with 24 nodes accessing the same channel and demonstrated that, in a high traffic set-up, the measured improvement was up to 5.8 in comparison with the Pure-ALOHA measured throughput. This solution requires the implementation of a different medium access protocol and, therefore, it is not compatible with current LoRaWAN networks.

In order to meet the requirements of the real-time traffic management in Industrial IoT Applications, Leonardi et. al. [6] worked on a solution based on a centralized MAC scheme. In this scheme, a central node synchronized all end-nodes broadcasting a periodic beacon. After a beacon, the time to send information is divided in two periods—the first is intended for non-periodic unconfirmed data in which end-nodes use the ALOHA mechanism for channel access, and the second is intended for periodic real-time confirmed data flows in which end-nodes access the channel with a TDMA mechanism. This solution seems to be devised for periodic transmissions in the contention-free period, guaranteeing no collisions, high reliability and bounded end-to-end delays. However, in the content-access period (with the Pure ALOHA medium access strategy), reliability is not ensured for the non-periodic transmissions.

In [7] a synchronization and scheduling solution is proposed, in which a central entity assigns the communication slots to the end-nodes based on several policies and infrequent signaling messages. In this assignment, it is necessary to know the traffic periodicity and the clock accuracy of end-nodes, so the central entity can assign the required time slots. The limitation of this work is the requirement of periodic traffic, otherwise the central entity will not know the number of time slots to assign.

Another time-slotted based solution is presented in [8], where it is computed a hash algorithm with the nodes’ addresses to assign them a specific time slot to transmit. The network server leverages the last slot sent in the broadcast frame in order to handle the acknowledgements and time synchronization at the expense of extra energy cost.

To achieve energy-efficiency and increase the network performance, the work in [9] designs a new LoRaWAN network architecture and a new MAC protocol based on unicast and broadcast TDMA communications. These communications are started by a sink and an intermediate entity, the cluster head, is responsible for the rely of wake-up beacons to synchronize the end-devices and avoid collisions. This solution needs the implementation of a non-standard LoRaWAN transceiver.

The different solutions proposed in EXPLoRa [10] are useful when taking into account different traffic types from different applications or in situations in which there are areas with different traffic demands requiring a balanced resource allocation. However, this solution requires a centralized entity that has to monitor and manage periodically the network behaviour.

FCA-LoRa [11] achieves a fairness resource allocation with collision avoidance. It is based on the broadcasting of periodic beacon frames sent by the gateways. This novel MAC protocol uses an alternative version of the CSMA/CA algorithm for listening the medium before the transmission of a packet (instead of using the ALOHA protocol used in original LoRaWAN).

The main differences between these works and our proposal are the following: (1) our proposal is compatible with current LoRaWAN networks since it works on top of the existing MAC protocol, and (2) after the initial LoRaWAN join procedure, no extra signaling or communication with the end-device is required.

## 3. Technical Overview of LoRa/LoRaWAN

The physical layer of a LoRaWAN network is called LoRa. LoRa is a chirp spread spectrum based modulation developed by Cycleo in 2009 and acquired by Semtech in 2012. An advantage of the LoRa modulation is the low complexity of the receiver design due to the equivalent offsets in timing and frequency between the transmitter and the receiver. The data signal is modulated onto a chirp signal that increases or decreases its frequency with time. The chirp rate defines the spectral bandwidth (*BW*) of a LoRa signal, meaning that for example, a spectral *BW* of 125 kHz corresponds to a chip rate of 125,000 chips/s. Assuming a fixed *BW*, the data rate can change depending on the employed spreading factor (*SF*). SF (which varies between 7 and 12) is the number of raw bits carried per symbol. Thus, the data rate $R_{b}$ is calculated as follows:

(1) $$R_{b}=SF\times \left(\frac{BW}{2^{SF}}\right)\times \left(\frac{4}{4+CR}\right),$$

where the first term is the spreading factor, the second term corresponds to $R_{s}$ or symbol rate (symbols/s) and the third term depends on the coding rate (CR) (The LoRa modulation includes a FEC (Forward Error Correction) scheme in order to find and correct erroneous bits by adding redundancy in the code, improving the robustness of the transmitted signal [12]. CR is the Code Rate and its values can vary between 1 and 4). Thus, assuming fixed BW and coding rate, the data rate decreases as the SF increases.

**Figure 1:**
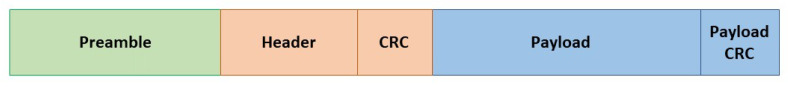
LoRa packet format.

The information is then transmitted following the packet format defined in Figure 1.

The Time on Air (ToA) or the duration of the packet transmission is given by the sum of the preamble and payload duration:

(2) $$T_{packet}=ToA=T_{preamble}+T_{payload}.$$

The preamble is common to all modem configurations. Its duration is given by the following expression:

(3) $$T_{preamble}=\left(n_{preamble}+4.25\right)T_{sym}.$$

where $n_{preamble}$ is the number of programmed preamble symbols and $T_{sym}$ is the symbol duration which can be defined as the necessary time to send $2^{SF}$ chips at the chip rate. $T_{sym}$ is calculated with Equation (4).

(4) $$T_{sym}=\frac{2^{SF}}{BW}.$$

The payload duration is given by Equation (5). The payloadSymbNb is defined as the number of symbols that form the header and the packet payload and it is calculated with Equation (6), where PL is the payload length, *H* is equal to 0 when the explicit mode of the header is enabled and 1 otherwise (When explicit mode is disabled, the header is removed because the properties of its fields are fixed.) and DE corresponds with the low data rate optimization in order to increase the robustness of the LoRa link at low data rates, so that, it is enabled for SF≥11.

(5) $$T_{payload}=payloadSymbNb\times T_{sym}$$

(6) $$payloadSymnNb=8+max(ceil\left(\frac{8PL-4SF+28+16-20H+1}{4(SF-2DE)}\right),0).$$

Table 1 presents a data rate configuration assuming a 125 kHz bandwidth and the default LoRaWAN Code Rate (4/5). The bit rate increases and the *ToA* decreases as the *SF* is decremented.

**Figure 2:**
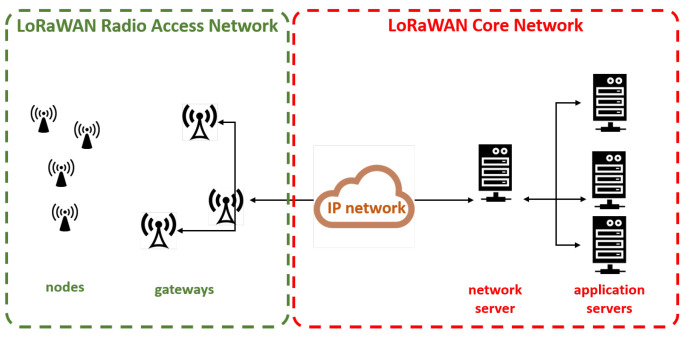
LoRaWAN network architecture.

The LoRaWAN open standard defines the network architecture and describes the communication protocol to allow the connection between each entity of the network. The protocol and network architecture have a big influence on determining the battery lifetime of a node, the network capacity, the security, and the variety of applications that can be served by the network [13]. A typical LoRaWAN network (see Figure 2) consists on a star-of-stars topology where one or more gateways (concentrators) relay download and upload traffic between a variety of end-nodes (that may be either mobile or mounted at a fixed location) and a central network server respectively. The network server manages the traffic between each end-device and the associated application server. The communication between end-devices and gateways consists of single-hop LoRa or FSK connections, whereas gateways make use of standard IP connections to the network server. All the communications are bi-directional.

The communication between end-nodes and gateways takes place using different channels and spreading factors. The transmission channel is chosen in every transmission by the end-device in a pseudo-random way and the SF (or DR) is selected taking into account the communication range and message duration through an Adaptive Data Rate (ADR) scheme [14]. The DR for each transmission is chosen taken taking into account the link budget (the higher the link budget, the faster DR or SF selected). Simultaneous transmissions in the same channel but in different SFs will never collided due to the orthogonality of the SFs. So that, this feature can be used to improve the network capacity.

The LoRaWAN standard defines three classes of LoRaWAN end-devices. The implementation of Class A is mandatory for all devices. This class is the most employed due to its low energy consumption. In Class A devices, two receiving windows are opened after an uplink transmission in order to receive a downlink transmission. The uplink transmissions in this class are scheduled using an ALOHA scheme, that is, the end-device transmits every time it has pending data without using any listen-before-talk mechanism. Class A devices are typically used for sensor nodes which are battery operated. Class B devices work similar to Class A devices, but extra receive windows are opened at scheduled times using beacon frames. This class is typically used for battery operated actuators. Class C devices are continuously listening except when transmitting, that is, the receive window is not closed. Class C is intended for mains powered actuators since continuous reception requires more power.

In Europe, LoRaWAN networks operates in the EU863-870 ISM band which allows up to eight channels with a 125 kHz bandwidth each, being three of them mandatory (868.1, 868.3, and 868.5 MHz). In order to respect ETSI regulations, LoRaWAN devices are limited to a duty-cycle lower than 1%. Moreover, there is not dwell time limitation in this band.

### Limitations of the LoRa MAC

This section presents some studied limitations of this protocol that significantly influence on the network capacity.

As mentioned, LoRaWAN employs a light MAC protocol based on P-ALOHA for scheduling uplink transmissions from end-devices. It is a simple multiple access protocol in which, every time the end-device has data to transmit, a packet is sent without any link coordination. Thus, if two or more nodes transmit data simultaneously, a collision will occur. The higher the number of end-devices accessing the network, the higher the number of collisions. Hence, this situation increases the packet error rate (PER), resulting in the degradation of the performance and the reduction of the network capacity.

Sundaram et al. [15] analyzes several research problems in LoRa networks such as energy consumption, communication range, multiple access, security, and so forth, and identifies many current works which try to provide appropriate solutions to mitigate these problems. One of the problems which significantly affects the performance of a LoRaWAN network is the medium access, which is related to the link coordination and the resource allocation in order to handle collisions and allocate reasonable resources to end-devices based on the deployed environment.

Adelantado et al. [4] highlights the importance of the coordination in a single shared infrastructure running many applications with different requirements (in terms of reliability, maximum latency etc.) using an ALOHA-based access.

These LoRaWAN MAC challenges are being investigated and there are some proposed solutions adapted to different scenarios which will be described below.

## 4. LoRaWAN MAC Proposal

We propose a resource allocation algorithm with the aim of reducing the number of collisions and therefore increasing network capacity. This solution, called Collision Avoidance Resource Allocation for LoRaWAN networks (CARA), allows end-devices to access the medium with minimal collisions. CARA is based on a distributed scheduling of channels and SFs at the LoRaWAN MAC layer of the end-devices.

The Pure-ALOHA medium access protocol is still maintained as detailed in the LoRaWAN specification. The end-devices use this protocol to access the medium, therefore, every time a device has a pending packet to transmit, it will wait for a random amount of time, and, after this period of time, it will send the packet with a certain channel and SF without previously listening to the medium.

Taking advantage of the orthogonality of the SFs, the channels × SFs space is divided so each pair of values represents an independent “resource block”. Different end-nodes can simultaneously transmit in different resource blocks without colliding.

Taking into account that the ISM frequency band in Europe allows up to 8 LoRa 125 KHz channels configuration and there are 6 different spreading factors, there are a total of 48 orthogonal resource blocks $RB_{j},j\in \left\{1,48\right\}$. Without loss of generality, we assume that the first 6th resource blocks utilize the first channel and spreading factors from 7 to 12, the second 6th RBs utilize the second channel and spreading factors from 7 to 12, and so on. Each node will divide the time in windows with a duration of $T_{window}$. If node *i* transmits during the *k*-th window, that is, $t\in \{t_{0}+k\times T_{window},t_{0}+\left(k+1\right)\times T_{window}\}$, it will utilize a specific resource block $RB_{i,k}$.

In order to compute which resource block (i.e., channel and spreading factor) has to be used for transmission, that is, $RB_{i,k}$, the gateway associated to a set of motes will be responsible for assigning to node *i* (1) a set of eligible spreading factors $\bar{SF_{i}}$ and (2) the initial resource block $RB_{j_{i}}$ for a reference time $t_{0}$. $\bar{SF_{i}}={\left\{s_{i,7},\ldots ,s_{i,j},\ldots s_{i,12}\right\}}^{T}$ is a binary vector which represents the spreading factors available to a node *i*, where $s_{i,j}\in \left\{0,1\right\}$ indicates whether this particular spreading factor can be used or not. With these parameters, the resource block selected by node *i* in the *k*-th window (starting from $t_{0}$) is given by Equation (7).

(7) $$RB_{i,k}=RB_{\hat{j}}|\hat{j}=\left(j_{i}+k\right)\;\mathrm{mod}\;\left(8\times \Sigma_{l=7}^{12}s_{i,l}\right),$$

where $\hat{j}\in \left\{1,8\times \Sigma_{l=7}^{12}s_{i,l}\right\}$, that is, only the available spreading factors ($s_{i,\hat{j}}=1$) are considered.

**Figure 3:**
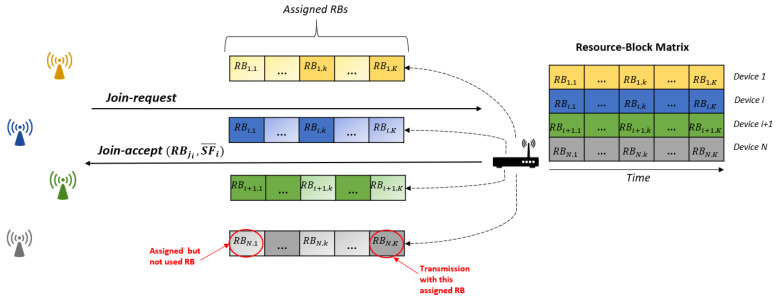
Join scheme in Collision Avoidance Resource Allocation (CARA) with the assignment for the assignment of resource blocks.

**Figure 4:**
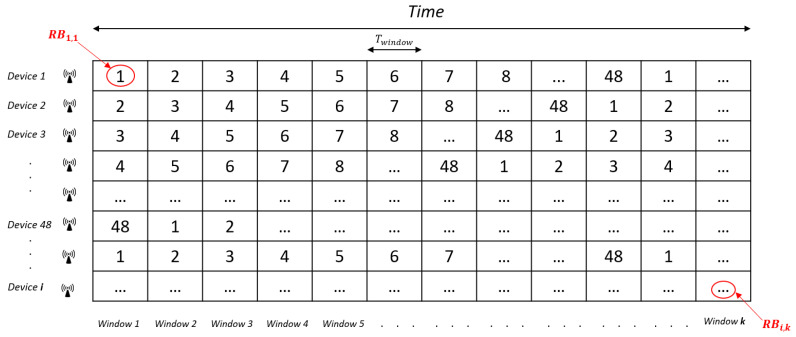
Resource block matrix $RB_{matrix}$.

The procedure for assigning resource blocks to end-devices is shown in Figure 3. When a device wants to join a LoRaWAN network, it will send a “Join-Request” message to the gateway. For each new device that joins the network, the gateway reserves a row of the resource block matrix (depicted in Figure 4). The value of this matrix in the *i*-th row and the *k*-th column is $RB_{i,k}$, that is, the resource block that node *i* will use in window *k*, that is, $RB_{matrix}=\left(\begin{array}{ccccc} RB_{1,1} & \ldots & RB_{1,k} & \ldots & RB_{1,K}\\ & & \ldots & & \\ RB_{i,1} & \ldots & RB_{i,k} & \ldots & RB_{i,K}\\ & & \ldots & & \\ RB_{N,1} & \ldots & RB_{N,k} & \ldots & RB_{N,K} \end{array}\right)$. The reserved row can be used by the device to compute the corresponding resource block for a specific window. To generate the row, the device only needs the initial resource block $RB_{j_{i}}$ and the usable spreading factors $\bar{SF_{i}}$.

Both parameters are calculated by the gateway in order to uniformly distribute the devices among the different resource blocks, and are sent in the “Join-Accept” message. If required, for example, due to changing radio conditions or network load, these parameters may be sent at any time for class B and class C devices or in ACK messages for class A devices (i.e., after transmitting a data frame).

To avoid collisions between devices, we use a cyclical shift between each row. As a result, two devices *a* and *b* with $RB_{j_{a}}\neq RB_{j_{b}}$ will never collide (in the case of both devices using all SFs). These can be easily seen in Figure 4, in which two rows with different initial values (column 1) will never have the same value in the same column. This is the reason why our scheme highly reduces the number of collisions, being null if the number of devices is lower than the number of resource blocks (48).

**Figure 5:**
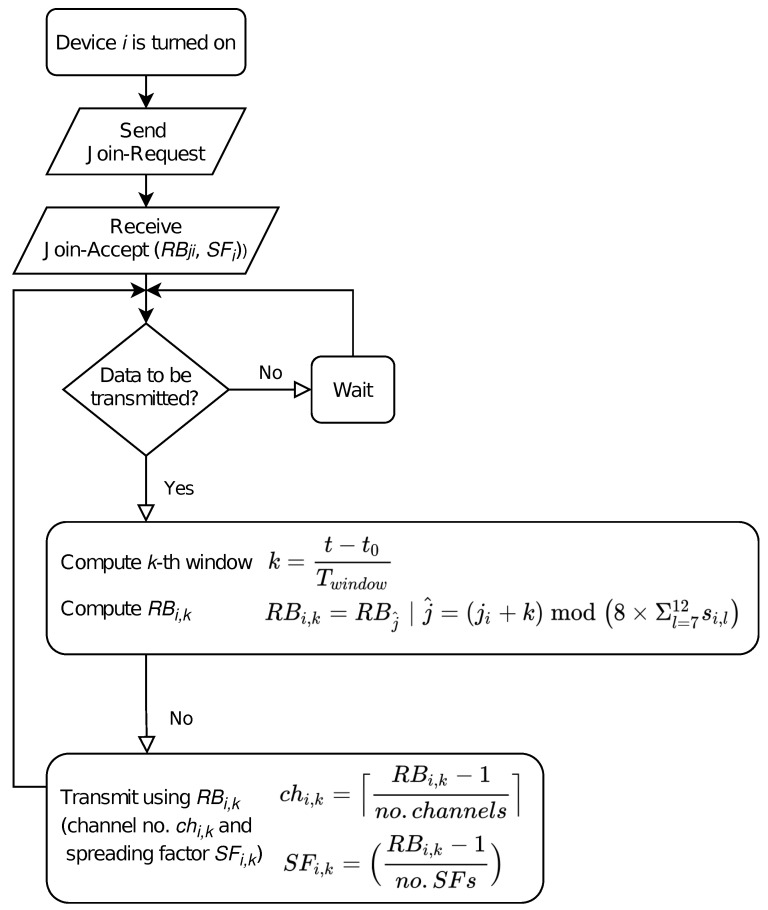
CARA flowchart.

For the sake of readability, we also include in Figure 5 a flowchart which describes the procedure from the node’s perspective.

CARA requires synchronization among devices. For such purpose, the *DeviceTime* MAC commands [14] may be used, which provides a worst case accuracy of ±100 ms. GPS clock synchronization may also be used if available on devices. If required, for example, to avoid problems due to the accuracy of the time synchronization, a guard time at the end of each window may be added (e.g., 100 ms when using *DeviceTime* MAC messages).

### Extension to Avoid Border Effects

**Figure 6:**
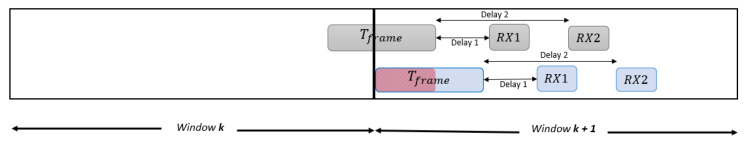
Border effect between time windows.

Even if two devices transmit in different resource blocks in the same window, it may occur that one of the transmissions finishes during the next time window and produce a collision. For example, in Figure 4 device 2 may start transmitting in the first window ($RB_{2,1}=2$) but may finish this transmission in the second window, which may cause a collision if device 1 start transmitting in the second window ($RB_{1,2}$ = 2). An example is shown in Figure 6.

## 5. Experimentation and Performance Results

In this section we first introduce the system model and the main assumptions used in our LoRaWAN simulator. Then, the simulator is validated against the theoretical model from [16]. We also verify that the simulator exhibits null collisions when CARA is tested with 48 or less nodes, that is, all devices are assigned different resource blocks. Finally, we evaluate the performance of CARA for different scenarios and we also detailed the main pros of our solution and its limitations.

### 5.1. System Model and Simulation Setup

**Figure 7:**
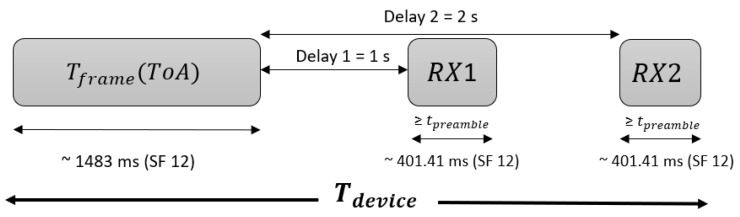
T${}_{device}$ scheme.

With the aim of assessing the effectiveness of our modified LoRaWAN MAC layer, we have implemented our proposal in a MATLAB-based dynamic simulator. Next we explain the main assumptions that we have considered for the implementation:

- End-devices are fixed.
- Each mote transmits frames with a 24-byte payload in non-acknowledged mode and meets the duty cycle restriction for the EU868-870 MHz ISM band (<1%).
- For a successful transmission, no other device can transmit in the same resource block while the transmitter sends the frame and waits for a possible ACK during both the first and the second RX windows. Otherwise, a collision occurs. Since the duration time of each reception window is usually $T_{preamble}$ and the time to open each reception window is one second, this time can be calculated as $T_{device}=T_{frame}+2\times T_{preamble}+2$ s. In the worst case, with SF12, this means that $T_{device}=2.62\times T_{frame}$. This is shown in Figure 7.
- We assume that there is a traffic source with an infinite number of devices that collectively form an independent Poisson source with an aggregate mean packet generation rate of λ packets/s [17]. The interarrival time between the transmissions of each device is independent and exponentially distributed. We can denote the $RB_{n,k}$ offered traffic as G ($RB_{n,k}$) = λ($RB_{n,k}$) · T${}_{device}$.
- The probability that an arbitrary scheduled packet in a specific channel is successfully transmitted, can be calculated with Equation (8). Since LoRaWAN employs several channels (or RBs in the case of CARA), the system throughput can be computed as the addition of the total throughput for each RB (Equation (9)).
  (8) $$P_{s}=\frac{\#succeedpackets}{\#(succeedpackets+collidedpackets)}$$
  (9) $$S_{total}=\sum_{n=1}^{N}\sum_{k=1}^{K}S\left(RB_{n,k}\right)=\sum_{n=1}^{N}\sum_{k=1}^{K}G\left(RB_{n,k}\right)\cdot P_{s}\left(RB_{n,k}\right).$$
- Table 2 includes relevant parameters that have been considered in the simulation.

In order to apply this novel resource block allocation scheme in realistic scenarios, a propagation model has been implemented assuming an urban environment called “Macro Cell Propagation Model”, which is based on [18,19].

The signal power that the gateway receives from each mote is calculated with the following expression:

(10) $$S_{RX}\left(dBm\right)=S_{TX}\left(dBm\right)+G_{TX}\left(dBi\right)+G_{RX}\left(dBi\right)-L_{prop}\left(dB\right)-X_{\sigma }\left(dB\right),$$

where:

− $S_{TX}$ is the signal power that a mote transmits (usually 14 dBm).
− $G_{TX}$ and $G_{RX}$ refer to the transmitter and receiver antenna gains, respectively.
− $L_{propagation}$ represents the urban environment path loss assuming that all the building heights are similar [19] and is calculated according to Equation (11). $Dh_{b}$ and *R* are the gateway height in meters and the distance between the end-device and the gateway in kilometers, respectively. *f* is the operating frequency in MHz (in our case we assume 868 MHz). A log-normally distributed shadowing with a standard deviation of 10 dB is also added ($X_{\sigma }$).

(11) $$L=40\cdot \left(1-4\cdot 10^{-3}\cdot Dh_{b}\right)\cdot \log_{10}\left(R\right)-18\log_{10}\left(Dh_{b}\right)+21\cdot \log_{10}\left(f\right)+80dB.$$

The considered scenario consists of a gateway located in the origin of coordinates. Motes have been positioned in a randomly manner following a uniform distribution around the aforementioned gateway.

The resource block allocation is carried out taking into account the information in Table 3. According to the strength of the signal received by the gateway, it will assign a more robust SF, such as SF10, SF11 or SF12, when the signal is weak. On the contrary, if the received signal is strong, a faster SF will be employed, for example, SF7, SF8 or SF9. Furthermore, it is assumed that there is no inter-SF interference.

### 5.2. Simulator Validation

We first design a LoRaWAN standard simulator in order to first validate its satisfactory operation.

**Figure 8:**
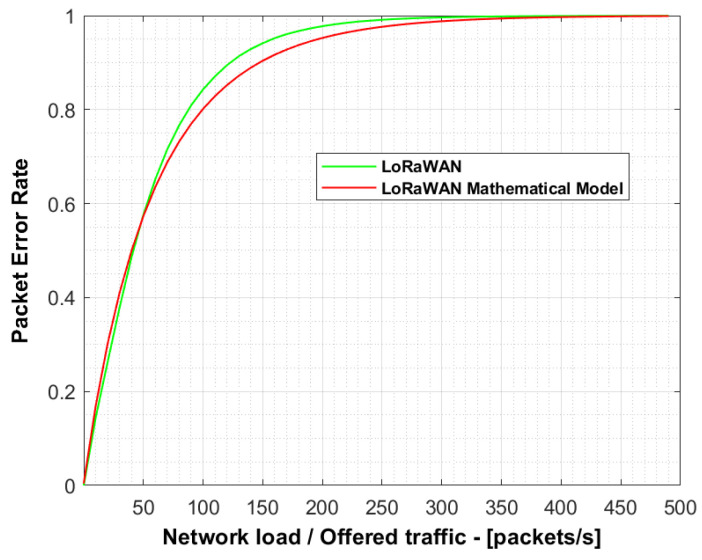
Comparison of packet error rate (PER) for 50,000 devices between results from simulations assuming a standard LoRaWAN network and from the theoretical mathematical model.

Figure 8 depicts the packet error rate for 50,000 devices with the objective of verifying the simulator behaviour compared with to the mathematical model proposed in [16].

The green and red lines represent the results from simulations assuming a standard LoRaWAN network and from the aforementioned mathematical model, respectively.

Both results are similar, albeit there is a little difference owing to the mathematical model considering an infinite number of nodes. The maximum relative error is 3.98% for a network load of 150 packets/s, which validates the correct behaviour of our simulator.

Afterwards, we modified the simulator with the new LoRa MAC proposal without first considering the propagation model depicted in Section 5.1, that is, assuming ideal propagation conditions. As an initial verification, we checked that the proposed design allows the simultaneous transmission of up to 48 devices without the existence of collisions. For this purpose, a simulation with exactly 48 motes generating traffic has been carried out.

**Figure 9:**
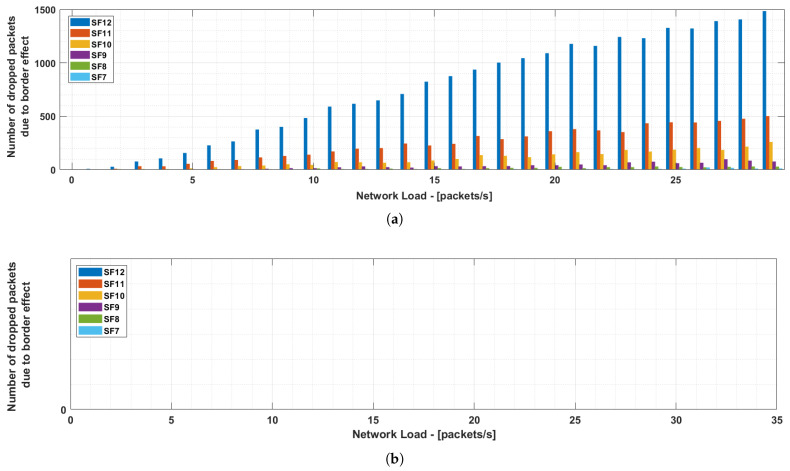
Collisions (**a**) with no border effect check and (**b**) avoiding the border effect.

Figure 9a shows the number of collisions for each spreading factor with different network loads. Analysing the simulations, all the collisions were due to the border effect. These collisions mainly occurs in SF12 since the higher ToA of the transmissions increases the probability of collisions. As an example, in a simulation with a network load of 30 packets/s, 51,222 packets were sent and only 2365 collided (PER = 4.6%). 1485 of these packets were transmitted with SF12 (62.8% of the collisions).

For this reason, the simulator was later modified so that nodes will avoid transmissions spread over two different time windows (i.e., starting on window *k* and finishing on window k+1). If this were to happen, nodes would postpone their transmissions to the beginning of the next window. As shown in Figure 9b, when our solution to avoid the border effect is executed, the collision probability is null as expected.

### 5.3. Performance Evaluation of the Proposed Solution

In our first set of simulations, we compare a standard LoRaWAN network and a LoRaWAN network using our CARA scheme. We assume that 50,000 nodes are deployed and radio link conditions are ideal.

**Figure 10:**
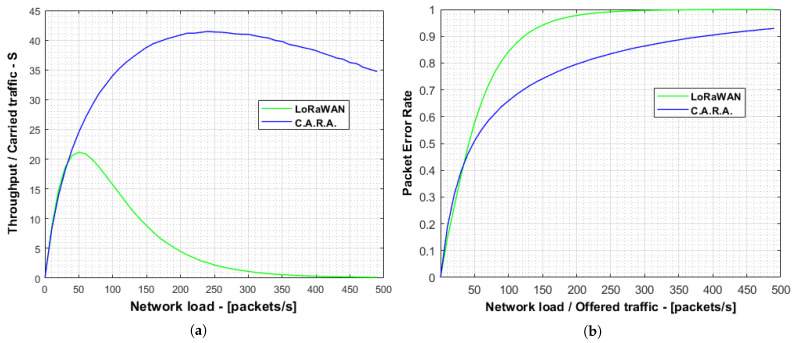
Comparison of the throughput and PER for 50,000 devices between a standard LoRaWAN network and using CARA. (**a**) System throughput and (**b**) Packet Error Rate.

As shown in Figure 10, our proposal outperforms the standard LoRaWAN achieving a maximum PER gain of 30.1% at a network load of 150 packets/s (PER was 71.4% in our proposal and 92.9% in standard LoRaWAN). Since this first set of simulations assumes ideal radio conditions, the packet errors are due to the collisions meaning that our solution significantly reduces the collision probability. This PER reduction allows the system to increase the maximum capacity (system throughput) from 21 packets/s to 41 packets/s, producing a relative gain of 95.2%.

The second set of simulations considers a realistic urban scenario assuming the propagation model explained in Section 5.1.

Two campaigns of simulations have been carried out with 1000 devices placed in the scenario. In the first campaign, the SF assignment have been carried out according to the ADR algorithm proposed in LoRaWAN standard [20]. Therefore, since we assumed fixed devices, the SF assigned to each device depends on the received sensitivity.

In the second campaign, devices use the SFs in which they can transmit. For example, if a device can transmit in SF9, it will hop between SF9, SF10, SF11 and SF12. Additionally, the size of the scenario is also modified in order to understand the effect of the coverage area on the performance and SF usage distribution. We differentiate results for “fast devices” (those whose lower eligible SF is SF7, SF8 or SF9) and “slow devices” (those whole lower eligible SF is SF10, SF11 or SF12). Table 4 shows the simulated areas (from 2 km × 2 km up to 10 km × 10 km) and the distribution of “fast devices” and “slow devices” according to the propagation model and the gateway sensitivity (see Table 3). As expected, a smaller area has a higher percentage of “fast devices” since they are closer to the gateway and therefore the radio conditions are better. On the contrary, a larger area means that the percentage of “slow devices” increases due to the same reasoning.

**Figure 11:**
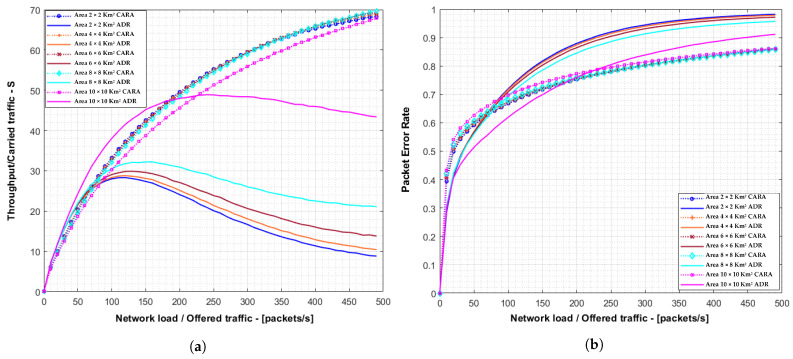
Throughput and PER for 1000 devices in the case of non groups of SF with different area dimensions. (**a**) Mean throughput of the system for ADR and CARA cases. (**b**) Packet Error Rate of the system in ADR and CARA cases.

Figure 11a,b represent the mean system throughput and PER for each campaign of simulations using coverage areas between 2 × 2 km${}^{2}$ to 10 × 10 km${}^{2}$. It can be seen that, for areas between 2 × 2 km${}^{2}$ and 8 × 8 km${}^{2}$, the CARA algorithm in this realistic scenario decrements the packet error ratio, achieving a maximum throughput up to almost 40% higher than the maximum achieved with ADR. However, with an area of 10 × 10 km${}^{2}$ we can observed a reduction of the average throughput for medium/low network loads using CARA. As the coverage area grows, the number of “slow devices” also increases. This is common for both CARA and standard LoRaWAN. However, using CARA, “fast devices” also utilize slow SFs part of the time. This produces a concentration of network load being carried over slow SFs, causing a higher number of collisions. This effect is more noticeable with larger areas due to the spreading factors distribution. On the contrary, when the coverage area is smaller, there are more devices using cyclically all SFs. In this case the SF distribution is more uniform and, thus, this effect is negligible and the improvement for using CARA is higher.

In light of the obtained results some conclusions can be highlighted. The first one is that with CARA it is obtained a great overall network throughput up to an area of 8 × 8 km${}^{2}$ with 1000 devices. For longer areas, due to the less proximity of the end devices to the gateway, the slowest and more robust data rates are more frequently used and the probability of collisions increases. On the contrary, with the same number of end devices but smaller areas, CARA contributes to the increase of the throughput and presents a great improvement compared to the throughput obtained with the ADR algorithm. Another result to highlight is that, although our solution slightly increases the overall transmission time and may lead to a little increase in energy consumption compared to the ADR algorithm proposed in the LoRaWAN specification, it achieves a fairer resource sharing. For example, in a coverage area of 2 × 2 km${}^{2}$ the ADR algorithm assigns shortest SFs due to the proximity of the end devices to the gateway, thus allowing shortest transmission times. However, this leads to more collisions due to the saturation of the fastest RBs. With the CARA algorithm, owing to the set of SFs each device is able to use, it leads to a more uniform RBs utilization, causing less collisions and therefore improving the overall network throughput.

## 6. Coexistence of LoRaWAN with CARA

A key aspect to consider is the coexistence between CARA and traditional LoRaWAN devices. In order to assess the performance of a LoRaWAN network with a mixture of devices, a new campaign of simulations has been carried out. We employed three set of simulations with different distribution between traditional LoRaWAN and CARA devices. The first set included 25% of LoRaWAN devices and the remaining 75% were CARA devices. In the other sets, the distributions were 50–50% and 75–25%, respectively. These simulations deployed 1000 devices assuming ideal radio link conditions (i.e., PER matches the collision probability).

**Figure 12:**
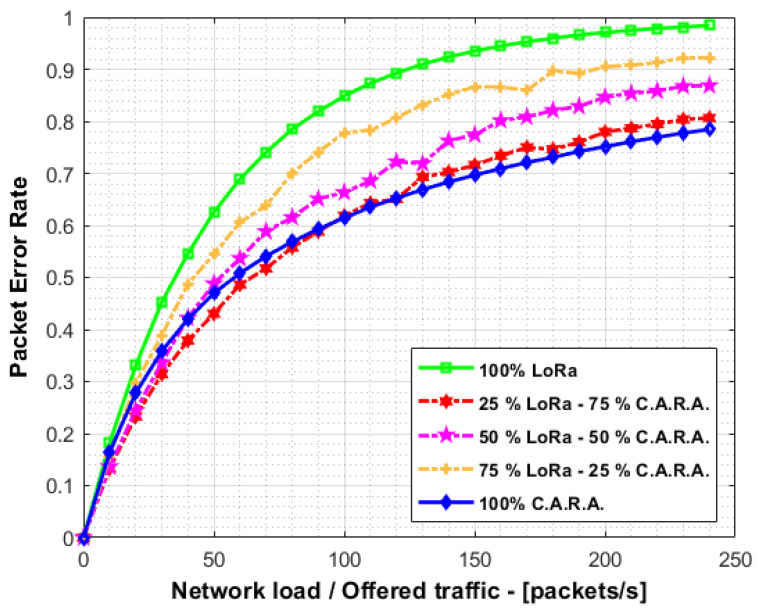
PER comparison coexistence LoRaWAN vs. C.A.R.A.

Figure 12 shows the aggregate packet error rate for the different distributions of devices. It is also included the cases with 100% of LoRaWAN devices and 100% of CARA devices. As shown, the collision probability decreases gradually as the percentage of CARA devices increases. This demonstrate that both device types can coexist in the same network.

## 7. Proof-of-Concept

As a proof of concept, we implemented CARA in an experimental testbed, part of the demonstrator developed for project A-TIC-241-UGR18 “Architecture for IoT networks oriented to environmental sustainability”, funded by the Andalusian Knowledge Agency.

Our testbed consists of 30 nodes connected to one LoRaWAN gateway, which in turn is also connected to the corresponding LoRaWAN network and application servers. The nodes are based on the Pycom’s FiPy development board [21], which is a multi-technology board (supporting Wi-Fi, BLE, LoRaWAN, SigFox and LTE-M/NB-IoT) with an ESP32 SoC. The LoRaWAN gateway is an IMST Lite Gateway [22], which contains a Raspberry Pi connected to an IMST ic880A LoRaWAN concentrator for the ISM 868 MHz band. We employed the Chirpstack platform [23], which provides open-source components for the LoRaWAN networks (network server, application server, gateway bridge, among others).

**Figure 13:**
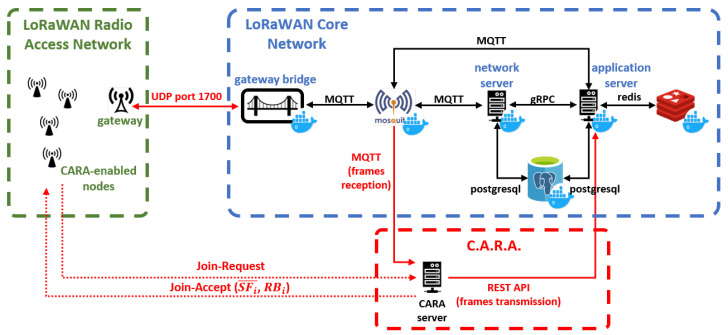
CARA architecture.

Figure 13 presents the network architecture for our proof-of-concept. As shown, a LoRaWAN network was deployed using the aforementioned nodes, gateway and servers. On top of this network, we implemented a CARA server, which is responsible of assigning the initial resource blocks for each node that joins the system. In addition, we also modified the firmware of the nodes in order to include the signaling handling and the resource block selection for each transmission. Since we do not have access to the source code of the MAC layer in the nodes, we implemented the required signaling at the application layer.

The CARA server is programmed using Python 3, and implements:

- An **MQTT listener** (subscriber) for messages from the application server. This allows the CARA server to receive signaling from nodes, that is, the Join-Request messages.
- A **signaling handler** which generates Join-Accept messages as a response for the received Join-Request frames. For that purpose, it uses the Chirpstack network server RESTful API [24]. This handler communicates with the resource assignment logic to obtain the available spreading factors ($\bar{SF_{i}}$) and the initial resource block ($RB_{i}$) for the node, parameters included in the Join-Accept.
- A **database client** to store and retrieve the current status of the CARA algorithm from a Postgresql database. In this database we store, for each node, its identifier (DevEUI), the initial resource block, the spreading factors that the node has available (in the form of a binary SF mask, in which 1 means that the SF can be used, 0 otherwise), and the time when the node was last seen (in order to remove that node from the CARA calculations after a configurable time without receiving data from that node).
- The **CARA resource assignment logic**, which checks which is the resource block with lowest assignments to be employed for the next node, considering which SFs are available.

Nodes are programmable using Micropython with the Pycom’s LoRa API [25]. We modified the devices’ firmware to include the following functionality (source code available at [26]):

- Since we do not have access to the source code of the MAC layer in order to include DeviceTime MAC commands, **node synchronization** is achieved by using the NTP protocol through a Wi-Fi connection.
- **Experiment parameters** are also sent over Wi-Fi to customize the testbed nodes.
- Nodes connect to the network using Over the Air Activation (**OTAA**). Activation By Personalization (ABP) has also been successfully tested.
- **CARA signaling** has been implemented on the node’s side. As soon as the node joins the network, it sends a Join-Request message to the application server. After this, it waits the reception of a Join-Accept message with the initial RB and the available SFs. Both messages are sent as application frames. If a Join-Accept is not received after a configurable time, the Join-Request is retransmitted.
- After this signaling, we assume a random time between packets for **traffic generation**. This time is composed of a fixed part (to avoid sending messages too fast) and a random part (to avoid correlation between transmissions from different nodes).
- For transmission, a **border effect check** has been implemented based on the time to initiate transmission and the ToA calculation (see Equation (2)). If the transmission ended on the next time window, it would be postponed until the beginning of that window.

**Figure 14:**
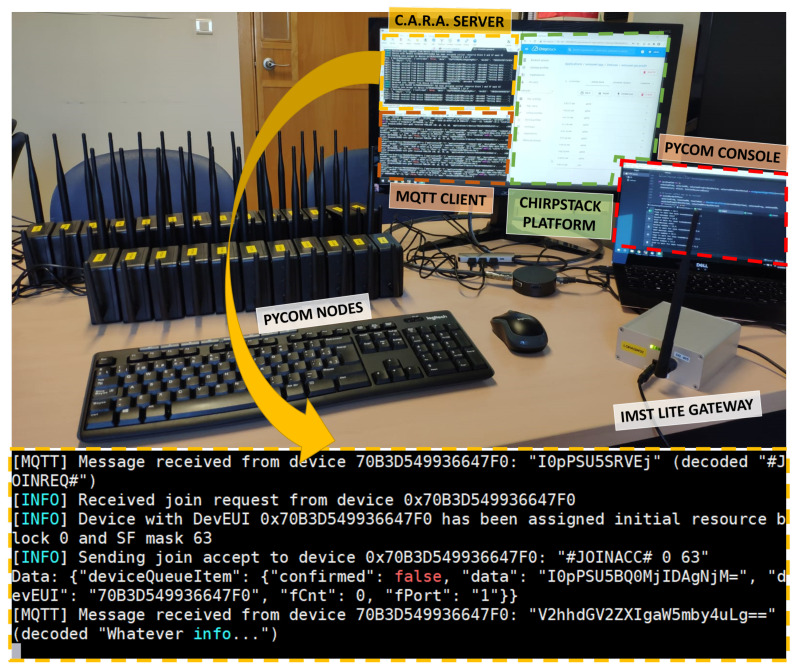
Testbed with our CARA implementation.

Figure 14 shows our testbed, which is composed of 30 nodes, one gateway and one server. The server implements both the Chirpstack platform and our CARA server. An example of the messages exchanged between one node and the CARA server is also provided. As shown, the CARA server receives one “#JOINREQ#“ message (*JoinRequest*), computes the initial resource block to be assigned to the node, and sends back this value in the “#JOINACC#” (*JoinAccept*) message. In addition, an spreading factor mask is also sent, which indicates which SFs are available to the node (one bit per SF, so 63 means that all the six SFs are available).

**Figure 15:**
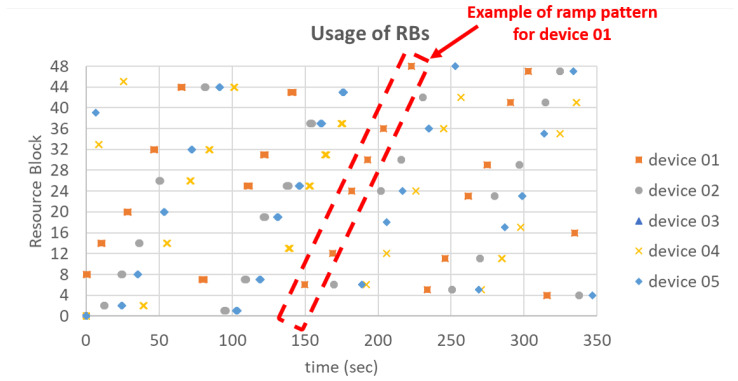
Resource blocks usage in the proof-of-concept (only 5 nodes shown).

In this experiment we have assumed that all spreading factors are available to all nodes. Data transmissions from each node are sent after 10+rand(10) s, where the rand(x) function returns a random value between 0 and *x*. This produces a network load of 6000 packets/s. As in our simulations, data frames contain 24 bytes. We have verified that, since the number of devices is smaller than the number of resource blocks, each device is granted a different initial resource block and therefore no collisions occur. Figure 15 shows an example of RBs allocation to the nodes on our testbed. Only 5 nodes are shown for the sake of clarity. As expected, nodes do not collide (residual collision probability lower than 0.5% due to other LoRaWAN devices in the surroundings) and the RBs are allocated sequentially to each node, following a ramp pattern. In addition to the CARA server log, we also checked the correct behaviour of our solution analyzing the traces from the node (in the Pycom console) and the frames received by the Chirpstack application server.

## 8. Conclusions and Future Work

In this paper, a collision avoidance resource allocation algorithm, named CARA, has been designed with the objective of increasing the capacity of LoRaWAN networks. CARA divides the wireless medium capacity into resource blocks which are defined by one channel and one spreading factor. Thanks to the orthogonality of the spreading factors in LoRa, transmissions in different resource blocks will not cause collisions. In addition, CARA leverages the existing joining procedure for parameters exchange and synchronization, not requiring communication between the end-devices and the network afterwards. CARA has been implemented in a MATLAB-based dynamic simulator, using a cyclical assignment which ensures that nodes with a different initial resource block will not collide. Simulation results show that our solution outperforms standard LoRaWAN networks achieving a 95.2% of capacity gain when radio link conditions are ideal. In realistic propagation environments, CARA increases the capacity by 40%. The coexistence between each type of devices has been also verified. Finally, a proof-of-concept has been developed using commercial equipment, which has allowed us to check the feasibility and the proper operation of the proposed solution.

Future work will include an experimental deployment in real urban and rural scenarios, analysing the performance of CARA in these environments. Additionally, the definition of a smart resource assignment algorithm that considers the RBs utilization in dense LoRaWAN networks is also left for further work.

## Figures and Tables

**Table 1:** Data Rate configuration for a 125 kHz bandwidth in the EU868 ISM band.

Data Rate	Spreading Factor	Bit Rate (bps)	ToA for a 24 billion Packet (ms)
0	12	293	1482.75
1	11	540	823.30
2	10	980	411.65
3	9	1757	205.82
4	8	3125	113.15
5	7	5470	61.70

**Table 2:** Simulation parameters.

Parameter	Value
BW	125 KHz
Code Rate	4/5
Payload Size	25 Bytes
Preamble length	8 symbols
SFs	(7–12)
Simulation time	1 h
Frequency band	868 MHz

**Table 3:** Gateway Sensitivity for each SF.

SF	Gateway Sensitivity (dBm)
12	−142.5
11	−140
10	−137.5
9	−135
8	−132.5
7	−130

**Table 4:** Number of devices transmitting in each group.

Area	Number of “Fast” Devices	Number of “Slow” Devices
2 × 2 km${}^{2}$	658	342
4 × 4 km${}^{2}$	646	354
6 × 6 km${}^{2}$	632	368
8 × 8 km${}^{2}$	623	377
10 × 10 km${}^{2}$	562	438

## Data Availability

The data presented in this study are available on request from the corresponding author.
